# Cultural Intelligence: What Is It and How Can It Effectively Be Measured?

**DOI:** 10.3390/jintelligence10030054

**Published:** 2022-08-05

**Authors:** Robert J. Sternberg, Ilaria Siriner, Jaime Oh, Chak Haang Wong

**Affiliations:** 1 Department of Psychology, Cornell University, Ithaca, NY 14853, USA; 2 Department of Psychology, University of Innsbruck, Innrain 52, 6020 Innsbruck, Austria; 3 Department of Higher Education, Teachers College, Columbia University, New York, NY 10027, USA

**Keywords:** culture, cultural intelligence, general intelligence, intelligence, maximum-performance test, practical intelligence, tacit knowledge, typical-performance test

## Abstract

We administered both maximum-performance and typical-performance assessments of cultural intelligence to 114 undergraduates in a selective university in the Northeast of the United States. We found that cultural intelligence could be measured by both maximum-performance and typical-performance tests of cultural intelligence. Cultural intelligence as assessed by a maximum-performance measure is largely distinct from the construct as assessed by a typical-performance measure. The maximum-performance test, the Sternberg Test of Cultural Intelligence (SCIT), showed high internal consistency and inter-rater reliability. Sections with problems from two content domains—Business (SCIT-B) and Leisure (SCIT-L) activities—were highly intercorrelated, suggesting they measured largely the same construct. The SCIT showed substantial correlations with another maximum-performance measure of cultural intelligence, Views-on-Culture. It also was correlated, at more modest levels, with fluid intelligence and personal intelligence tests. Factorially, the (a) maximum-performance cultural intelligence tests, (b) typical-performance cultural intelligence test and a test of openness to experience, and (c) fluid intelligence tests formed three separate factors.

## 1. Introduction

Cultural intelligence is one’s ability to adapt when confronted with problems arising in interactions with people or artifacts of cultures other than one’s own (Sternberg et al. 2021a). Some might view cultural intelligence as merely a special case of general intelligence, but there is at least some evidence that cultural intelligence is a distinct construct that is related but nonidentical to general intelligence (Ang et al. 2006, 2007, 2015, 2020; Sternberg 2008; Sternberg and Grigorenko 2006; Sternberg et al. 2021a; Van Dyne et al. 2008).

At least conceptually, there are three ways in which cultural intelligence might plausibly differ from general intelligence while at the same time being related to it:

First, cultural intelligence would seem to have a practical, tacit-knowledge-based component that makes it akin to what sometimes is called “practical intelligence”, which (arguably) is at least somewhat distinct from general intelligence (Hedlund 2020; Polanyi 1976; Sternberg and Hedlund 2002; Sternberg and Horvath 1999). Tacit knowledge is acquired from experience. It is a matter of not how much experience one has but rather of what one learns from that experience. Presumably, cultural intelligence, like crystallized intelligence, develops in part as a result of experience. Where it is more like practical intelligence than like crystallized intelligence is that it is procedural. It is not a matter of knowing *declarative* general information or vocabulary (as measured on the Wechsler intelligence scales, e.g., Wechsler (1944)) but rather like the *procedural* practical skills measured through situational judgment tests (Weekley and Ployhart 2005). Cultural intelligence is not something one can memorize, such as a vocabulary list or a set of facts. Rather, it is something that one deploys according to the intricacies of a situation and in light of the task and the persons involved.

Second, cultural intelligence can be seen as having metacognitive, cognitive, motivational, and behavioral components (Ang et al. 2006, 2007, 2015). The first two components are measured by tests of general intelligence, but in abstract contexts that are different from those of intercultural interactions. To the extent that intelligence comprises an interaction of person by task by situation (Sternberg 2021a), the metacognitive and cognitive components may be quite different from those that are displayed in a conventional intelligence testing situation. The metacognitive component is used, for example, to understand how one is thinking about the situation one is in—as friendly, hostile, indifferent, or whatever. The cognitive component is used to figure out what to do in the situation. The motivational component is used to create engagement with the situation—some people simply shy away from intercultural situations or refuse to accept them as involving norms potentially different from their own. Moreover, the behavioral component is used to enact the behavior one views as appropriate in a given situation.

Third, to be measured fully, one might wish to use a combination of typical-performance and maximum-performance measures. Past research suggests the two kinds of measures assess different aspects of cultural intelligence (Sternberg et al. 2021a), much as do typical- and maximum-performance measures of emotional intelligence (Rivers et al. 2020). There is, of course, no perfect measure of anything: Any measure has error built into it. For example, typical-performance measures are subject to deception, directed both against the tester and the test-taker (who may, for example, have an inflated perception of their own skills). They are also subject to bias—people use rating scales differently, so some tend to rate higher than others, much as would be true in grading in school. However, maximum-performance measures are also susceptible to bias, for example, how alert one happens to be at the time of testing and how one handles what are usually timed, multiple-choice, or short-answer tests.

Although maximum-performance and typical-performance tests sometimes are pitted against each other—as though one is the “correct” kind of test and the other an “incorrect” kind of test, or one is a better kind of test and the other is a lesser kind of test (Kunzmann 2019; Webster 2019)—we view them more as complementary than as competing. That said, a risk of self-report measures is that individuals simply do not know where they stand, or worse, that individuals who are low performers have greatly inflated perceptions of their own performance, the so-called Dunning–Kruger effect (Kruger and Dunning 1999). This is a particular problem in the study of wisdom, where epistemic humility is an essential component of wisdom (Grossmann et al. 2020), so more wise people often think of themselves as less wise and less wise people think of themselves as more wise.

It is not difficult to imagine the extension of the effect of cultural intelligence, wherein people of low cultural intelligence may view the problem of intercultural interaction as a simple problem of the people of perceived “inferior” cultures needing to adapt to those of perceived “superior” cultures; in contrast, the culturally intelligent person likely would realize that cultures differ considerably and cannot simply be placed on some kind of value scale from better to worse. Lest this sound exaggerated, it is worth remembering that the early history of psychology and especially of cross-cultural psychology is replete with examples of white male researchers from Western cultures imposing what they saw as their “superior” values on members of what they saw as “inferior” cultures (see, e.g., Gasquoine 1997; Gould 1981).

Why is cultural intelligence even important? The first and main reason is that intercultural interactions are omnipresent, whether we wish them to be or not. Countries can clash because they do not understand each other’s values, as can individuals and groups (Markus and Conner 2014). For example, what is viewed as acceptable behavior in male–female interactions differs widely across cultures (Wood and Eagly 2002). Intercultural interactions, for many people, are no longer exotic or some kind of luxury. They have become a nearly inevitable part of everyday life.

There is a second reason cultural intelligence is important, however. Regardless of how it affects our interactions with people of other cultures, it increases our understanding of our own cultures. Presuppositions and cultural patterns that once may have seemed to be necessary parts of life may now be seen as merely single options among many different options. Men who have grown up in a culture that shows a general disrespect for women, however, and who may not have thought that there were other viable options, may now see that the way their culture treats women is not a necessity or even perhaps desirable—it is a choice and perhaps a suboptimal one.

A third reason for the importance of cultural intelligence is that, for whatever arguments one might make in one direction or another about the teachability of general intelligence, cultural intelligence is clearly teachable at some level. No one is born with cultural intelligence. They may be born with propensities at one level or another. However, tacit knowledge is acquired from experience (Sternberg and Hedlund 2002). To the extent that it can be isolated from experience, it can be taught. People can learn from their experience and any instruction they receive about how to interact better with people of diverse cultures. Moreover, they may even increase their own self-understanding and self-awareness.

In a previous study, Sternberg et al. (2021a) studied cultural intelligence and how to measure it. In the current study, we used what is now called the Sternberg Cultural Intelligence Test (SCIT), which comprises two subscales, a Business subscale (SCIT-B) and a Leisure subscale (SCIT-L). There were certain aspects of that earlier study that, at least in principle, could be improved upon, and that we addressed in the current study. In particular:

First, the subscale coefficient alpha internal consistency reliabilities of .79 (SCIT-B) and .77 (SCIT-L), with a combined reliability of .87, were somewhat lower than would have been ideal. One might have hoped for subscale reliabilities over .80 and total reliability over .90. We therefore lengthened the measures in the hope of attaining higher internal consistency reliability and possibly higher validity as well, as a larger sampling of behavior would have been considered. The subscales, which were 10 and 9 items in length, were here each 12 items in length in the current study. This is an increase in length of 20% for the SCIT-B and 25% for the SCIT-L.

Second, the instructions in the earlier version of the SCIT did not make clear that the items would be scored in such a way that multiple solutions to a problem would result in a higher score. The idea was that, in intercultural interactions, the first response sometimes is ineffective or, at best, only partially effective. Some participants may not have realized that multiple responses were desired. In this revised version, participants were informed that they should “come up with a solution to solve the problems in scenarios and alternative solutions if the main one does not work out”. This change may have increased both validity and reliability.

Third, the previous study lacked, we believe, sufficient measures for convergent validation. In particular, there was a need for questions that were relevant to cultural intelligence and that would correlate with SCIT scores. To increase the number of hoped-for convergent validators, we introduced a Views-on-Culture measure that would measure knowledge and skills that we believed would be relevant to cultural intelligence.

Fourth, the previous study had just 6.4% Black or African American participants. Our hope in the present study was to have greater representation of Black participants. Indeed, in this study, the representation was 13.2% of the participants.

Fifth, the test graders in the previous study also devised the rubric for grading. One could have argued—and a reader did argue—that the higher inter-rater reliability was because the raters devised the rubric under which they were rating. The three raters in the current study used an enhanced version of the previous rubric and hence did not devise it themselves, so that the inter-rater reliability figure could not be due to their having devised the rubric themselves. Nevertheless, we obtained high inter-rater reliabilities for the SCIT—inter-rater reliability amongst the three graders resulted in the reliability values of .97 for the SCIT-B, .96 for the SCIT-L, and .98 for the SCIT-B+SCIT-L. The rubric for this work was more detailed than for the previous work.

Sixth, in the previous study, we had two graders. In the current study, we had three graders in order to increase inter-rater reliability.

Thus, in this study, we sought both to show the replicability of the earlier results in terms of the construct validity of the SCIT for measuring cultural intelligence and also to refine past work to address some of the inadequacies of the previous work, as described above. In particular, based on the past research, we hypothesized that the maximum-performance tests would correlate with each other, and the typical-performance tests would correlate with each other, but the maximum-performance tests would not correlate much, if at all, with the typical-performance tests. This pattern derives from the notion that the two types of tests measure relatively distinct aspects of cultural intelligence. We further expected the maximum-performance tests of cultural intelligence to show factorial loadings different from those of fluid intelligence tests. 

## 2. Method

### 2.1. Participants

A total of 114 undergraduate and graduate students attending a selective university in the Northeast of the United States participated in the data collection, which was conducted through an online survey. Of these participants, 93 of them were female, 20 of them were male, and 1 of them indicated “other.” The average age of the participants was 20.19 years with a standard deviation of 1.03. The self-indicated racial/ethnic composition was 34.2% Asian and Asian American, 29.8% White or Caucasian, 13.2% Black or African American, 9.6% Hispanic or Latino, 1.8% American Indian or Alaska Native, and 7.9% of two or more races; 3.5% preferred not to answer.

### 2.2. Materials

There was a total of 9 assessments in the form of an online survey, administered through Qualtrics. These assessments consisted of two psychometric assessments, which included (1) Letter Sets and (2) Figure Classification; two maximum-performance Sternberg Cultural Intelligence Test subtests, (3) one of which detailed a scenario pertaining to a business trip and (4) the other of which depicted a leisure trip; a (5) Views-on-Culture questionnaire we created that was composed of 3 items; a (6) typical-performance Cultural Intelligence Scale (CQS—Van Dyne et al. 2008); an (7) Openness to Experience (OE) scale (Johnson 2014); a (8) test of Personal Intelligence Mini (TOPI—Mayer et al. 2018); and a (9) demographic questionnaire we constructed.

**Psychometric Assessments.** The two psychometric assessments administered for this study were (1) Letter Sets and (2) Figure Classification. The Letter Sets test required participants to rule out one letter set that did not fit in with the four other sets given. The Figure Classification test required participants to select and categorize each given figure into a group based on feature similarity. The purpose of the psychometric assessments was to measure fluid intelligence. The tests were taken from the Kit of Factor-Referenced Cognitive Tests (Ekstrom et al. 1963). This section was scored based on how many correct answers were given, with each correct answer yielding one point.

**Maximum-Performance Sternberg Cultural Intelligence Test (SCIT).** Two subtests of the maximum-performance Sternberg Cultural Intelligence Test were developed. These two versions and the test as a whole were a modification of the test presented in Sternberg et al. (2021a). One subtest was related to a business trip (SCIT-B) and the other subtest was related to a leisure trip (SCIT-L). In both subtests of the Sternberg Cultural Intelligence Test, one could find a variety of simulated, realistic scenarios that one might experience in different cultural contexts. Participants must say what they would do to overcome and deal with certain challenges presented when traveling to a new cultural environment. 

The general instructions were:

“Instructions: Please read the following information and come up with a solution to solve the problems in scenarios and alternative solutions if the main one does not work out.”

For example, in the SCIT-B, a business executive travels to a foreign country with which the executive has little familiarity to try to reach an important business agreement.

“You have just arrived on your current confidential assignment in a foreign country with which you are largely unfamiliar. Your assignment is to negotiate a memorandum of agreement between your organization and a large organization in the foreign country. You were told that you were expected to return to the US with a signed agreement. Before leaving the US, you were given very little information about your destination country, and most of that was basic information on the political system, imports and exports, and the general economy. You do not know the language and you know that relations with the country are tense. You realize that your room in the hotel in which you are staying has no access to the World Wide Web. Moreover, your cell phone does not work in this country.”

One item from the SCIT-B was:

“As you get ready to approach customs at the airport, a woman seems to come out of nowhere and approaches you. You think you recognize her from your trip. You can’t quite place her but believe she was one of the employees of the organization with which you negotiated. She says that the organization forgot to give you a farewell gift and that she was instructed to give it to you before you departed. She has only now caught up with you. She shoves a gift box into your hands. It is packed in gift wrap with a gold ribbon but otherwise has no identifying marks. On one hand, you don’t want to insult the organization but, on the other hand, you have no idea what is in the box. What would you do?”

There were 12 items each for both the SCIT-B and for the SCIT-L. Each item was graded by three different graders. The final score was calculated by the average of all three graders. The scoring was based on a 5-point scale, in which 1 indicated a poorly answered item and 5 indicated a thoroughly and elaborately answered item. The grading rubric for the Sternberg Cultural Intelligence Tests (both SCIT-B and SCIT-L) was as follows:

*Zero points*: No answer/blank.

*One point*: Provided one plausible response with no/vague further explanation, for example, “I would go to a hospital.”

*Two points*: Provided one or two plausible responses with some explanations, for example, “I would go use hand gestures to indicate my illness and ask for a map to find a hospital.”

*Three points:* Provided two or more plausible responses with more elaborated explanations, for example, “I would first do…then…if something went wrong, I would….”.

*Four points*: Provided three or more plausible responses with elaborated explanations, for example, “I would use nonverbal body language to show that my stomach is in pain. If there was a pharmacy nearby, I would point that out to a local and then use nonverbal body language to see if the local could help me find the hospital. If that did not work, I would pretend to be listening to someone’s heartbeat with a stethoscope and see if someone could help me find a hospital after that.”

*Five points*: Provided three or more plausible (and novel/unique) responses with specific and detailed explanations.

There were no 5’s given in grading this sample. The highest score given was a 4, with the lowest score a 1.

The inter-rater reliabilities, computed as intraclass correlations coefficients, amongst the three graders resulted in reliability values of .97 for the SCIT-B, .96 for the SCIT-L, .98 for the SCIT-B + SCIT-L, .78 for Views-on-Culture Item 1, .76 for Views-on-Culture Item 2, and .86 for Views-on-Culture Item 3. The relatively robust inter-rater reliabilities may be attributed in part to the establishment and careful implementation of a standard rubric. The three graders were themselves somewhat diverse: Two were Asian-Americans and one was German. All were thoroughly trained on the rating protocol.

**Views-on-Culture (VC) (3 items).** The Views-on-Culture (VC) questionnaire consisted of three items we created, each intended to gauge each participant’s interests and personal opinions regarding different aspects of culture.

We included this measure for convergent validation because previous work, we believed, showed the need for an additional measure to demonstrate convergent validity. At this time, there were no fully validated maximum-performance measures of cultural intelligence. Ideally, we would have used an already existing, if not fully validated and standardized, maximum-performance measure of cultural intelligence, based on the notion of measurement dealing with unexpected situations in a novel cultural setting. However, we were unable to find any adequate existing convergent validator at all. Schwarzenthal et al. (2019) devised a situational judgment test, but it was for students meeting students of other cultures, which was situationally quite distinct from our measure. The measure we used, in contrast, was designed for adult, post-student use to measure cultural intelligence in business and leisure settings. Another possibility would have been the measure of Rockstuhl et al. (2015), but we had 1 ½ hours of testing time and we did not have the remaining testing time available for a test longer than the one we used. 

Each of the three items of the Views-on-Culture measure is listed below:

**Item 1**: “Some people believe it is worthwhile to learn to speak at least one foreign language fluently. Other people believe it is not worthwhile.

*(a)*  *What do you believe?*
*(b)*  *Give the reasons why you believe what you believe.”*

**Item 2**: “Some people believe it is worthwhile spending a significant amount of time (at least six months) living in a foreign country. Other people believe it is not worthwhile.

*(a)*  *Have you ever lived in a foreign country for at least six months?*
*(b)*  *What do you believe?*
*(c)*  *Give the reasons why you believe what you believe.”*

**Item 3:** “You meet someone from a foreign country who, in a conversation, expresses beliefs with which you strongly disagree. You are surprised that they could believe and express such a thing.

*(a)*  *What would you say or do?*
*(b)*  *Why would you say or do that?”*

For grading, Part a of Item 1 was not given a score. Item 1, Part b, was graded on a 3-point scale based on the number of reasons judged as satisfactory given while assessing for quality, with 3 rated as a very good answer. Item 2, Part a, was graded with “yes” as 1 point and “no” as 0. Item 2, Part b, was not given a score. Item 2, Part c, was scored for quality on a scale of 0–3. Item 3, Parts a and b, were also both scored for quality on a scale of 0–3. The rubric for the items scored on a scale of 0–3 was as follows:

*Zero:* no answer/perverse answer (irrelevant/mean);

*One:* weak response (“I don’t understand why you would say that”);

*Two*: good answer;

*Three*: very good answer.

**Typical-Performance Cultural Intelligence Scale (CQS).** The typical-performance Cultural Intelligence Scale (Van Dyne et al. 2008) is presented as a measure of an individual’s capability of navigating various cultural settings that are different from their own. It is composed of 20 statements, one such example being, “I am conscious of the cultural knowledge I apply to cross-cultural interactions”. Each of the 20 statements was to be rated by the participant as a self-report, on a scale of 1 to 7, based on the following scale: (1 = strongly disagree; 2 = disagree; 3 = more or less disagree; 4 = undecided; 5 = more or less agree; 6 = agree; 7 = strongly agree).

The purpose of the CQS is to measure the individual’s cultural intelligence as typical performance. The CQS is composed of cognitive, motivational, and behavioral CQ subscales.

**Openness to Experience (OE).** The typical-performance Openness to Experience (OE) scale was modified from the Big Five Inventory Personality scale (Johnson 2014). In this section, a total of 24 statement items gauging individual personality traits and their resulting attitudes on life were shown. Participants were then asked to depict their level of agreement with each statement, on a scale of 1 to 5, with 1 representing a very inaccurate statement with regard to oneself and 5 representing a very accurate statement.

**Test of Personal Intelligence Mini-12 (TOPI).** The Test of Personal Intelligence was adapted from the full TOPI test, which is a questionnaire composed of 134 items (Mayer et al. 2018). The maximum-performance TOPI mini was a reformed version that was much shorter, intended for quick use in the laboratory. It was composed of 12 items that assessed the individual’s problem-solving capabilities. Participants were asked to answer and pick one correct answer of four choices given after reading a short passage.

**Demographic Questionnaire.** The demographic questionnaire requested information such as age, gender, ethnicity, year at the university, SAT and ACT scores, cumulative college GPA, and the number of different countries the participants had visited.

### 2.3. Design

The design of this study was correlational. The main dependent variables were scores on the Sternberg Cultural Intelligence Test (SCIT—including SCIT-B for Business items and SCIT-L for Leisure items). Other scores were used as independent variables to predict scores on the SCIT.

### 2.4. Procedure

This study was administered in the form of an online survey through the Qualtrics platform. Participants in this study were gathered through an online platform for students at the university. First, before taking the assessments, participants were asked to read and sign the consent form shown before any tests and surveys were administered. Upon signing and the approval of consent, they were then taken to the two psychometric assessments: Letter Sets and then Figure Classification. The psychometric sections were automatically timed and once the time limit was reached, the system forwarded the participant to the next section directly. The time limit for the Letter Sets was 7 min and for the Figure Classification was 8 min. The following sections, including the Sternberg Cultural Intelligence Test (SCIT-B, SCIT-L), Views-on-Cultures (VC) questionnaire, Cultural Intelligence Scale (CQS), Openness to Experience (OE) Scale, Test of Personal Intelligence Mini (TOPI), and the Demographic Questionnaire, all did not have a time limit. Upon the completion of the study, a written debriefing form was presented to the participants.

## 3. Results

### 3.1. Basic Statistics

Descriptive statistics for demographic questions (age, cross-cultural experience in years, and number of countries visited), OE, psychometric assessments (Letter Sets, Figure Classification, and TOPI), standardized admissions tests (ACT and SAT with subtests reading and math), as well as college GPA, are summarized in Table 1. Table 1 further provides basic statistics for the tools that were used to assess cultural intelligence: the maximum-performance Sternberg Cultural Intelligence Test (SCIT—including the Business and Leisure subtests SCIT-B and SCIT-L), the three items that assessed Views-on-Culture, and the typical-performance Cultural Intelligence Scale—CQS—by Van Dyne et al. (2008).

The SCIT overall mean was 50.50 with a standard deviation of 13.25. Mean ACT and SAT scores in our population were higher than the average population of college students with the national ACT average of 20.6 and the national SAT averages of the SAT Reading of 533 and the SAT Math of 527. Our values for our selective university sample were, for the ACT overall, 33.56, and for the SATs, 727.30 Reading, and 754.12 Math (https://nces.ed.gov/programs/digest/d17/tables/dt17_226.40.asp, accessed on 3 August 2022; https://www.number2.com/average-act-score/#What_is_the_National_Average_ACT_Score, accessed on 1 August 2022). Our sample also featured smaller standard deviations of 49.44 in SAT Reading and 56.63 in SAT Math, compared with the national standard deviations of 100 and 107. The national standard deviation for the ACT is 4.8, considerably greater than our standard deviation of 1.49. However, many participants did not take the standardized tests, and one can expect that those who did submit scores may well have scored on the higher side of the university population mean, had all students taken the tests.

### 3.2. Analyses of Variance

Table 2 summarizes the results of an analysis of variance for sex. None of the mean differences were significant. Results of the ANOVA for ethnicity are contained in Table 3. Our analysis revealed significant relationships of test scores with ethnicity for the SAT Reading (*p* < 0.05), SAT/ACT (*p* < 0.05), GPA (*p* < 0.01), OE (*p* < 0.05), and ethnicity. Black or African-born participants had lower overall scores in SAT Reading, SAT/ACT, and GPA compared to other ethnicities. Students who preferred not to answer their ethnicity had lower overall OE scores. There are many possible causes of such differences; we have no basis for choosing among them.

### 3.3. Internal Consistency Reliabilities

Table 4 provides the internal consistency reliabilities as measured by coefficient alpha. The Sternberg Cultural Intelligence Test (Business, Leisure, and total) showed high reliabilities (0.95, 0.94, and 0.97), with coefficient alpha reliabilities comparable to or higher than those for the other measures used. The results also were better than those of the previous version of the test (Sternberg et al. 2021a), which were 0.79, 0.77, and 0.87, respectively, perhaps because the test was revised and lengthened.

### 3.4. Intercorrelations

Table 5 shows the intercorrelations among all measures used in this study. 

First, significant correlations were not found between the Sternberg Cultural Intelligence Test and either self-reported standardized admissions tests (SAT/ACT) or self-reported GPA. Only the first Views-on-Culture item correlated significantly (*p* < 0.05, *r* = .28) with the SAT Reading score and also (*p* < .05, *r* = .23) college GPA (*p* < 0.05, *r* = .23). The fourth dimension of the CQS also correlated (*p* < 0.05, *r* = .28) with the college GPA as well as the ACT (*p* < 0.05, *r* = .26). However, correlations were generally rather weak.

Second, the Sternberg Cultural Intelligence Test (SCIT) showed significant correlations with the fluid intelligence measures: The Business, Leisure, and total scores and all Views-on-Culture items showed significant intercorrelations with the Letter Sets—(*r* = .31, *r* = .37, *r* = .35, *r* = .34, *r* = .24, *r* = .32)—all *p* < 0.01, except for Views-on-Culture Item 2, with *p* < 0.05. All correlations, except for the Views-on-Culture Item 2, showed medium effect sizes. The SCIT also correlated with the Figure Classification task (*r* = .20, *p* < .05; *r* = .25, *p* < .01; *r* = .23, *p* < .05; *r* = .28, *p* < .01; *r* = .26, *p* < .01), as did the Views-on-Culture items, except for the second item.

Third, the SCIT and all three Views-on-Culture items intercorrelated with the Test of Personal Intelligence (TOPI—*r* = .24, *r* = .31, *r* = .28, *r* = .38, *r* = .25, *r* = .29, all *p* < 0.01) at small to medium effect sizes. In contrast, there were no correlations between the typical-performance CQS and the Letter Sets, Figure Classification, or Test of Personal Intelligence, except for the second dimension of the CQS with the Test of Personal Intelligence (*r* = −.22, *p* < .05).

Fourth, the Business (SCIT-B) and Leisure (SCIT-L) tests correlated at high effect sizes with each other (which was expected, as all parts were developed to measure cultural intelligence) and with the three Views-on-Culture items (*p* < 0.01). The SCIT-B correlated with the SCIT-L at *r* = .85, with the first Views-on-Culture item at *r* = .45, with the second Views-on-Culture item at *r* = .46, and with the third Views-on-Culture item at *r* = .39. The SCIT-L correlated with the first and second Views-on-Culture items at *r* = .51 and with the third Views-on-Culture item at *r* = .47. The total score’s correlations with the first, second and third Views-on-Culture items were at *r* = .50, at *r* = .50, and at *r* = .44. The first Views-on-Culture item correlated at *r* = .46 with the second and at *r* = .54 with the third Views-on-Culture item. The second Views- on-Culture item correlated at *r* = .54 with the third Views-on-Culture item.

Fifth, in contrast to those statistically significant correlations, the maximum-performance SCIT did not correlate significantly with the typical-performance-based CQS, nor with its dimensional subscores. The Views-on-Culture items, however, did correlate with it: Item Number One showed correlations with the CQS (at *r* = .20, *p* < 0.05) and its first (metacognitive) and fourth (behavioral) dimensions (*r* = .21 and *r* = .19, both *p* < .05), the second item correlated with first and fourth dimensions as well (*r* = .21 and *r* = .19, both *p* < .05 as well), and the third item correlated with the motivational CQS dimension (*r* = .19, *p* < .05). The total SCIT score and SCIT-B score as well as all three Views-on-Culture items showed small- to medium-sized correlations (Cohen 1988) with OE (*r* = .22, *p* < .05; *r* = .20, *p* < .05; *r* = .28, *p* < .01; *r* = .27, *p* < .01; and *r* = .22, *p* < .05). Lastly, the first and third Views-on-Culture items correlated with the number of countries visited (each correlation, 0.19, *p* < .05).

### 3.5. Principal Component Analyses

Table 6 provides the rotated principal component analysis for the maximum-performance Letter Sets and Figure Classification, the two subscales (SCIT-B and SCIT-L) of the maximum-performance Sternberg Cultural Intelligence Test (SCIT), all three items of the Views-on-Culture measure, the typical-performance CQS, the typical-performance OE scale, and the maximum-performance TOPI. The measures used clustered based on three distinct groups in order of decreasing portions of variance are explained here:

(1) SCIT-B, SCIT-L, and VC items;
(2) Letter Sets, Figure Classification, and the TOPI;
(3) CQS and OE.

A principal component analysis for the psychometric measures, the total score of the SCIT measure, the Views-on-Culture (VC) measure, the CQS, and the Openness to Experience (OE) measure is compiled in Table 7. In analogy to the results in Table 6, the SCIT maximum-performance measures comprised the first principal component, the psychometric tests the second factor, and the typical-performance CQS and OE the third.

Table 8 shows the two subtests (Business and Leisure) of the Sternberg Cultural Intelligence Test, the three Views-on-Culture items, the CQS, the Openness to Experience (OE) scale, the Test of Personal Intelligence (TOPI), self-reported SAT/ACT scores, and college GPA, with four principal components constructed to account for most of the variance. Notably, while the two subscales of the Sternberg Cultural Intelligence Test and the Views-on-Culture items were featured as the first principal component mirroring the results in Table 6 and Table 7, the second component consisted of the standardized SAT/ACT test scores and the college GPA. The third component was the Test of Personal Intelligence and Openness to Experience, which was also contained in the fourth component alongside the CQS.

Table 9 provides the results of a principal component analysis of the total Sternberg Cultural Intelligence Test score, the Views-on-Culture items, the CQS, Openness to Experience, the Test of Personal Intelligence, the SAT to ACT conversion, and college GPA. Three components had Eigenvalues greater than 1: the Total SCIT score and the three VC items made up the first factor, SAT/ACT and college GPA the second, and Openness to Experience and the Test of Personal Intelligence the third component. The CQS did show major loadings on any factors.

Finally, Table 10 shows a principal factor analysis for the same tests with a very similar outcome as the results of the principal component analysis. In general, principal factor analyses revealed results quite similar to the principal component analyses.

## 4. Discussion

We sought, in this study, to continue and refine the construct validation of the Sternberg Cultural Intelligence Test (SCIT). The results largely replicate and extend the results of Sternberg et al. (2021a). Our main findings, largely consistent with previous research, were that:

1. The overall pattern of results, as described below, seems to suggest that cultural intelligence is a construct that can be measured by a maximum-performance measure with substantial reliability and validity.
2. Cultural intelligence, as measured by a maximum-performance measure, is somewhat different from cultural intelligence as measured by a typical-performance measure. The SCIT did not correlate significantly with the CQS, a typical-performance measure of cultural intelligence. Thus, the way people characterize themselves in intercultural situations is not related significantly to their maximum performance in at least some such situations. A maximum-performance measurement of cultural intelligence by the SCIT was reliable in terms of internal consistency. The internal-consistency reliabilities were .97 (SCIT), .95 (SCIT-B), and .94 (SCIT-L). Inter-rater reliability was also high (.97) for the SCIT.
3. The SCIT-B and the SCIT-L were highly intercorrelated, *r* = .85, *p* < .001, suggesting that the test measures a coherent set of skills across at least two domains—Business and Leisure activities.
4. The SCIT is not a disguised test of scholastic or academic achievement. It correlated significantly neither with self-reported standardized admissions test scores (SAT/ACT) nor with self-reported cumulative college school GPA.
5. However, the SCIT does relate to fluid intelligence, with significant correlations with Letter Sets in the .30s and significant correlations with Figure Classification problems in the .20s.
6. The SCIT does correlate significantly with a maximum-performance measure of Views-on-Culture, through which participants are asked about their views on (a) the importance of learning a foreign language, (b) the value of living abroad for at least six months, and (c) their views on how to resolve a discrepancy in values between them and a foreigner. The correlations of the SCIT with the three items, respectively, were *r* = .50, *r* = .50, and *r* = .44. Thus, the maximum-performance measures of cultural intelligence seem to show convergent validity with respect to each other.
7. The SCIT also correlated significantly with the TOPI (*r =* .28). Thus, the maximum-performance tests relevant to the socioculturally related aspects of intelligence were significantly correlated with each other.
8. Factorially, the maximum-performance cultural intelligence tests—the SCIT and the Views-on-Culture questions—factored together; the Letter Series and Figure Classification tests measuring fluid intelligence factored together; and the typical-performance CQS and Openness to Experience tests factored together.
9. Thus, the maximum-performance and typical-performance cultural intelligence tests showed external correlates, but with generally different measures. Both types of tests may measure somewhat different aspects of cultural intelligence.
10. Because we did a number of factor analyses with different and diverse variables included in the various analyses, which variables loaded where depended on the full set of variables that set up the context for each analysis. However, the results were consistent both across analyses and with regard to earlier work on cultural intelligence (Sternberg et al. 2021a). In the current work, the variables included were more diverse across factor analyses than those in the previous Sternberg et al. (2021a) work.

To conclude, first, we found here, as before, that the maximum-performance measures of cultural intelligence (the SCIT and the new measures in the present study, Views-on-Culture) loaded on the same factor. Maximum-performance cultural intelligence thus is replicated as a measure that appears to have integrity as a unified construct. These results suggest that the SCIT and the Views of Culture measure have at least some construct validity as converging measures of maximum-performance cultural intelligence, at the same time that future studies need to compare these measures with other existing maximum-performance measures, such as that of Rockstuhl et al. (2015).

Second, our measures of fluid intelligence—Letters Sets and Figure Classification—as in the previous work, loaded on the same factor, one measuring conventional fluid intelligence.

Third, SAT/ACT and cumulative college GPA consistently loaded on the same factor, suggesting a college preparedness/achievement factor that may have been akin to, but probably not identical to, crystallized intelligence. Both measured acquired academic knowledge and skills. (GPA was not included as a variable in the factor analyses of Sternberg et al. 2021a).

Fourth, the CQS, a typical-performance measure of cultural intelligence, never loaded on the same factor as the maximum-performance measures of cultural intelligence, as in Sternberg et al. (2021a). Maximum- and typical-performance cultural intelligence appear to be different constructs, one measuring cognitive aspects and the other more (self-reported) attitudinal aspects of cultural intelligence. This finding is similar to findings for wisdom (Kunzmann 2019; Webster 2019) and emotional intelligence (Rivers et al. 2020).

Fifth, where the CQS and Openness to Experience showed substantial factor loadings, they always loaded on the same factor, consistent with Sternberg et al. (2021a). In some analyses, there were not enough typical-performance measures to balance maximum-performance measures, so they did not both show substantial loadings.

Sixth and finally, the Test of Personal Intelligence (TOPI), a measure related to measures of emotional intelligence, showed somewhat variable patterns of factor loadings. It usually, but not always, loaded with Openness to Experience. Because we did not choose tests to study the construct validity of this measure, we cannot say definitively where it fit into the nomological net of our constructs and measures. Therefore, the loadings of this test were less stable than those of the previous work.

In terms of “improvements” on an earlier study (Sternberg et al. 2021a), (a) we substantially increased coefficient alpha internal consistency reliability, probably by making the SCIT longer and by clarifying the instructions, (b) we made clear to participants that we were seeking more than a single response to challenging intercultural situations, (c) we added the Views-on-Culture measure, which, as expected, provided convergent validation for the SCIT, (d) we more than doubled the percentage of participants who were African-American, although this change still left us with a sample restricted to college students from a selective university, and € we used a prior rubric from a previous cultural intelligence study (Sternberg et al. 2021a), rather than having the raters devise their own rubric. Most importantly, we largely replicated the past results.

As always, there are questions that remain unanswered. First, in our study, the individuals described in the SCIT were visiting a foreign country. However, many intercultural interactions occur when someone from a foreign country visits one’s own country. A more nearly complete test would have items in which an individual from a different culture visits one’s own culture, rather than vice versa. Second, our participants were all undergraduates from a selective Northeastern university. They were therefore not a representative sample from any population of interest. A more representative sample is needed. Third, it would be helpful to have performance-based measures of actual performances executed in intercultural contexts, as opposed to hypothetical situations presented on a computer. Fourth, it would help in future research more clearly to delineate the relationship between cultural intelligence, on the one hand, and social, practical, and emotional intelligence, on the other. Fifth, future research on our cultural intelligence measure should compare it to the Rockstuhl et al. (2015) measure and possibly the Schwarzenthal et al. (2019) measure as well. Finally, we need to learn more about the relationship between typical- and maximum-performance measures of cultural intelligence. As with measures of emotional intelligence (Rivers et al. 2020), typical- and maximum-performance measures seem to be measuring different things. How do they differentially relate to actual intercultural performances?

Cultural intelligence may once have been a luxury. People could grow up in their own little corners of the world and live and die there with few or no intercultural interactions. Such a life is becoming increasingly hard to lead. Moreover, cultural misunderstandings abound. It often is very challenging for people in one culture to understand why people in another culture think, feel, and act the way they do. Cultural intelligence provides an important key to unlocking the mysteries of what makes people different from us the way they are.

Although we believe our measure shows promise, until the measure is shown to predict actual behavior in real-world intercultural situations, its ecological validity as a measure of cultural intelligence cannot be comprehensively and fully demonstrated. This demonstration could be an important task for future research.

It would be easy but, we believe, mistaken to get into an argument over whether typical-performance measures such as the CQS “really” measure cultural intelligence or whether maximum-performance measures such as the SCIT do. No measure is perfect or complete. We adhere to the view expressed by Sternberg et al. (2021b) that intelligence has both typical- and maximum-performance aspects—that is, ones that are both attitudinal and ability-based. Moreover, in the end, while intellectual ability is important, how it is deployed, as determined by one’s attitudes, will determine how it affects adaptation to the environment (Sternberg 2021b). We believe the two kinds of measures in combination provide a better reading of a person’s cultural intelligence than either alone.

## Figures and Tables

**Table 1:** Mean scores and standard deviations.

	Mean	Standard Deviation	N
Age	20.19	1.03	113
ACT (MP)	33.56	1.49	61
SAT Reading (MP)	727.30	49.44	74
SAT Math (MP)	754.12	56.63	80
SAT to ACT conversion (MP)	32.83	2.26	103
Cumulative college GPA (MP)	3.74	0.29	109
Letter Sets (MP)	11.20	3.03	114
Figure Classification (MP)	66.91	21.58	114
SCIT-B (MP)	25.95	7.74	114
SCIT-L (MP)	24.56	6.04	114
SCIT total score (MP)	50.50	13.25	114
Views-on-Culture Item 1 (MP)	1.65	0.56	114
Views-on-Culture Item 2 (MP)	1.59	0.48	114
Views-on-Culture Item 3 (MP)	2.91	0.83	114
CQS (TP)	91.37	18.65	114
CQS Dimension 1 MC (TP)	21.61	3.61	114
CQS Dimension 2 COG (TP)	21.32	7.35	114
CQS Dimension 3 MOT (TP)	23.83	6.23	114
CQS Dimension 4 BEH (TP)	24.61	6.37	114
Openness to Experience (TP)	75.85	12.06	114
TOPI (MP)	9.99	2.18	114
CCE (demographic)	5.93	6.608	104
# of different countries visited	6.92	5.516	110

Note: MP = Maximum Performance; TP = Typical Performance; CQS (Van Dyne et al. (2008) ; the four dimensions of CQS are (1) Metacognitive, (2) Cognitive, (3) Motivational, and (4) Behavioral; TOPI = Test of Personal Intelligence; CCE = Cross-Cultural Experience in Years.

**Table 2:** Gender-based Analysis of Variance.

			Sum of Squares	df	Mean Square	F	Sig.
ACT * Gender	Between groups	(combined)	1.41	1	1.41	.63	.43
	Within groups		131.64	59	2.23		
	Total		133.05	60			
SAT Reading * Gender	Between groups	(combined)	3312.37	2	1656.18	.67	.51
	Within groups		175,147.09	71	2466.86		
	Total		178,459.46	73			
SAT Math * Gender	Between groups	(combined)	3137.06	2	1568.53	.48	.62
	Within groups		250,201.69	77	3249.37		
	Total		253,338.75	79			
SAT to ACT conversion * Gender	Between groups	(combined)	.85	2	.43	.08	.92
	Within groups		521.34	100	5.21		
	Total		522.19	102			
GPA * Gender	Between groups	(combined)	.05	2	.03	.31	.73
	Within groups		9.02	106	.09		
	Total		9.07	108			
Letter Sets * Gender	Between groups	(combined)	34.61	2	17.31	1.91	.15
	Within groups		1003.75	111	9.04		
	Total		1038.36	113			
Figure Classification * Gender	Between groups	(combined)	2429.08	2	1214.54	2.69	.07
	Within groups		50,178.05	111	452.05		
	Total		52,607.12	113			
SCIT-B * Gender	Between groups	(combined)	189.19	2	94.59	1.60	.21
	Within groups		6579.61	111	59.28		
	Total		6768.80	113			
SCIT-L * Gender	Between groups	(combined)	128.63	2	64.32	1.79	.17
	Within groups		3994.41	111	35.99		
	Total		4123.04	113			
SCIT Total * Gender	Between groups	(combined)	629.81	2	314.91	1.82	.17
	Within groups		19,219.13	111	173.15		
	Total		19,848.94	113			
VC Item 1 * Gender	Between groups	(combined)	1.17	2	.58	1.90	.16
	Within groups		34.15	111	.31		
	Total		35.32	113			
VC Item 2 * Gender	Between groups	(combined)	.07	2	.03	.14	.87
	Within groups		25.78	111	.23		
	Total		25.85	113			
VC Item 3 * Gender	Between groups	(combined)	1.45	2	.73	1.06	.35
	Within groups		75.72	111	.68		
	Total		77.17	113			
CQS * Gender	Between groups	(combined)	1131.68	2	565.84	1.65	.20
	Within groups		38,180.84	111	343.97		
	Total		39,312.53	113			
OE * Gender	Between groups	(combined)	120.34	2	60.17	.41	.66
	Within groups		16,302.12	111	146.87		
	Total		16,422.46	113			
TOPI * Gender	Between groups	(combined)	.99	2	.50	.10	.90
	Within groups		536.00	111	4.83		
	Total		536.99	113			

Note: SCIT-B = Cultural Intelligence − Business (Maximum Performance); SCIT-L = Cultural Intelligence − Leisure (Maximum Performance); SCIT = Overall Cultural Intelligence (Business and Leisure); VC = Views-on-Culture; CQS = Van Dyne et al. (2008) CQS (Typical Performance); OE = Openness to Experience; TOPI = Test of Personal Intelligence.

**Table 3:** Race/Ethnicity-based Analysis of Variance.

			Sum of Squares	df	Mean Square	F	Sig.
ACT * Ethnicity	Between groups	(combined)	17.19	6	2.87	1.34	.26
	Within groups		115.86	54	2.15		
	Total		133.05	60			
SAT Reading * Ethnicity	Between groups	(combined)	35,600.49	5	7120.10	3.39	.01
	Within groups		142,858.97	68	2100.87		
	Total		178,459.46	73			
SAT Math * Ethnicity	Between groups	(combined)	37,705.47	6	6284.24	2.13	.06
	Within groups		215,633.28	73	2953.88		
	Total		253,338.75	79			
SAT to ACT conversion * Ethnicity	Between groups	(combined)	82.93	6	13.82	3.02	.01
	Within groups		439.26	96	4.58		
	Total		522.19	102			
GPA * Ethnicity	Between groups	(combined)	1.71	6	.29	3.96	.00
	Within groups		7.36	102	.07		
	Total		9.07	108			
Letter Sets * Ethnicity	Between groups	(combined)	80.94	6	13.49	1.51	.18
	Within groups		957.42	107	8.95		
	Total		1038.36	113			
Figure Classification * Ethnicity	Between groups	(combined)	4464.75	6	744.13	1.65	.14
	Within groups		48,142.37	107	449.93		
	Total		52,607.12	113			
SCIT-B * Ethnicity	Between groups	(combined)	416.79	6	69.47	1.17	.33
	Within groups		6352.00	107	59.36		
	Total		6768.80	113			
SCIT-L * Ethnicity	Between groups	(combined)	223.78	6	37.30	1.02	.41
	Within groups		3899.26	107	36.44		
	Total		4123.04	113			
SCIT Total* Ethnicity	Between groups	(combined)	1182.25	6	197.04	1.13	.35
	Within groups		18,666.69	107	174.46		
	Total		19,848.94	113			
VC Item 1 * Ethnicity	Between groups	(combined)	1.34	6	.22	.70	.65
	Within groups		33.98	107	.32		
	Total		35.32	113			
VC Item 2 * Ethnicity	Between groups	(combined)	.91	6	.15	.65	.69
	Within groups		24.93	107	.23		
	Total		25.85	113			
VC Item 3 * Ethnicity	Between groups	(combined)	1.26	6	.21	.30	.94
	Within groups		75.91	107	.71		
	Total		77.17	113			
CQS * Ethnicity	Between groups	(combined)	1604.02	6	267.34	.76	.60
	Within groups		37,708.51	107	352.42		
	Total		39,312.53	113			
OE * Ethnicity	Between groups	(combined)	2027.00	6	337.83	2.51	.03
	Within groups		14,395.46	107	134.54		
	Total		16,422.46	113			
TOPI * Ethnicity	Between groups	(combined)	7.13	6	1.19	.24	.96
	Within groups		529.86	107	4.95		
	Total		536.99	113			

Note: SCIT-B = Cultural Intelligence − Business (Maximum Performance); SCIT-L = Cultural Intelligence − Leisure (Maximum Performance); SCIT = Overall Cultural Intelligence (Business and Leisure); VC = Views-on-Culture; CQS = Van Dyne et al. (2008) CQS (Typical Performance); OE = Openness to Experience; TOPI = Test of Personal Intelligence.

**Table 4:** Internal Consistency Reliabilities (Coefficient Alpha).

Test	Coefficient Alpha Reliability	Number of Items
Letter Sets	.81	15
Figure Classification	.96	112
Cultural Intelligence—Business (SCIT-B)	.95	12
Cultural Intelligence—Leisure (SCIT-L)	.94	12
SCIT Combined (SCIT-Total)	.97	24
CQS	.92	20
Openness to Experience	.84	24
Test of Personal Intelligence Mini (TOPI)	.72	12

**Table 5:** Intercorrelations.

		1	2	3	4	5	6	7	8	9	10	11	12	13	14	15	16	17	18	19	20	21	22
1	ACT	1	.55 **	.62 **	.93 **	.30 *	.02	.14	.07	.06	.07	−.06	−.02	.13	.19	.02	.11	.11	.26 *	−.18	−.03	.14	.09
2	SAT Reading	.55 **	1	.48 **	.79 **	.39 **	.15	.32 **	.16	.15	.16	.28 *	.10	.17	.10	.06	.07	.00	.16	.12	.10	.03	.11
3	SAT Math	.62 **	.48 **	1	.86 **	.26 *	.09	.27 *	−.14	−.15	−.15	.06	−.13	−.06	.06	.00	.13	−.07	.10	−.04	.04	.02	.07
4	SAT to ACT	.93 **	.79 **	.86 **	1	.34 **	.10	.25 *	.00	−.02	−.01	.10	−.09	.05	.08	.03	.06	−.01	.16	−.08	.10	−.01	.12
5	GPA	.30 *	.39 **	.26 *	.34 **	1	.29 **	.32 **	.02	.16	.09	.23 *	.17	.14	.11	.07	.06	.02	.21 *	.09	.01	−.16	.09
6	LS	.02	.15	.09	.10	.29 **	1	.57 **	.31 **	.37 **	.35 **	.34 **	.24 *	.32 **	.02	.05	−.07	.03	.06	.31 **	.49 **	−.12	−.03
7	FC	.14	.32 **	.27 *	.25 *	.32 **	.57 **	1	.20 *	.25 **	.23 *	.28 **	.08	.26 **	.09	.02	.15	.05	.04	.14	.20 *	.08	.10
8	SCIT-B	.07	.16	−.14	.00	.02	.31 **	.20 *	1	.85 **	.97 **	.45 **	.46 **	.39 **	.14	.10	.08	.09	.17	.22 *	.24 **	.07	.05
9	SCIT-L	.06	.15	−.15	−.02	.16	.37 **	.25 **	.85 **	1	.95 **	.51 **	.51 **	.47 **	.14	.10	.06	.12	.17	.17	.31 **	.03	.12
10	SCIT Total	.07	.16	−.15	−.01	.09	.35 **	.23 *	.97 **	.95 **	1	.50 **	.50 **	.44 **	.14	.10	.07	.11	.17	.20 *	.28 **	.05	.08
11	VC Item 1	−.06	.28 *	.06	.10	.23 *	.34 **	.28 **	.45 **	.51 **	.50 **	1	.46 **	.54 **	.20 *	.21 *	.07	.18	.19 *	.28 **	.38 **	.12	.19 *
12	VC Item 2	−.02	.10	−.13	−.09	.17	.24 *	.08	.46 **	.51 **	.50 **	.46 **	1	.54 **	.17	.21 *	.07	.12	.19 *	.27 **	.25 **	.15	.18
13	VC Item 3	.13	.17	−.06	.05	.14	.32 **	.26 **	.39 **	.47 **	.44 **	.54 **	.54 **	1	.18	.16	.10	.19 *	.13	.22 *	.29 **	.15	.19 *
14	CQS	.19	.10	.06	.08	.11	.02	.09	.14	.14	.14	.20 *	.17	.18	1	.74 **	.78 **	.81 **	.82 **	.26 **	−.05	.27 **	.25 **
15	CQS Dimension 1 MC	.02	.06	.00	.03	.07	.05	.02	.10	.10	.10	.21 *	.21 *	.16	.74 **	1	.40 **	.53 **	.61 **	.23 *	.07	.23 *	.10
16	CQS Dimension2 COG	.11	.07	.13	.06	.06	−.07	.15	.08	.06	.07	.07	.07	.10	.78 **	.4 0**	1	.46 **	.45 **	.20 *	−.22 *	.18	.18
17	CQS Dimension 3 MOT	.11	.00	−.07	−.01	.02	.03	.05	.09	.12	.11	.18	.12	.19 *	.81 **	.53 **	.46 **	1	.56 **	.29 **	.00	.23 *	.25 **
18	CQS Dimension 4 BEH	.26 *	.16	.10	.16	.21 *	.06	.04	.17	.17	.17	.19 *	.19 *	.13	.82 **	.61 **	.45 **	.56 **	1	.12	.06	.22 *	.2 3*
19	OE	−.18	.12	−.04	−.08	.09	.31 **	.14	.22 *	.17	.20 *	.28 **	.27 **	.22 *	.26 **	.23 *	.20 *	.29 **	.12	1	.28 **	.01	.07
20	TOPI	−.03	.10	.04	.10	.01	.49 **	.20 *	.24 **	.31 **	.28 **	.38 **	.25 **	.29 **	−.05	.07	−.22 *	.00	.06	.28 **	1	.04	.01
21	CCE	.14	.03	.02	−.01	−.16	−.12	.08	.07	.03	.05	.12	.15	.15	.27 **	.23 *	.18	.23 *	.22 *	.01	.04	1	.28 **
22	# Countries	.09	.11	.07	.12	.09	−.03	.10	.05	.12	.08	.19 *	.18	.19 *	.25 **	.10	.18	.25 **	.23 *	.07	.01	.28 **	1

* Correlation is significant at the 0.05 level (2-tailed); ** correlation is significant at the 0.01 level (2-tailed).

**Table 6:** Rotated Principal Components Matrix ^a^: Psychometric assessments, SCIT: Business (SCIT-B) and Leisure (SCIT-L), Views-on-Culture (VC) with three items, Cultural Intelligence Scale (CQS), Openness to Experience (OE), and Test of Personal Intelligence (TOPI).

	Component		
	1	2	3
Letter Sets (MP)	.21	.86	.06
Figure Classification (MP)	.06	.75	.09
Cultural Intelligence (MP)—Business	.84	.13	−.02
Cultural Intelligence (MP)—Leisure	.88	.19	−.04
Views-on-Culture Item 1 (MP)	.64	.32	.24
Views-on-Culture Item 2 (MP)	.74	.04	.23
Views-on-Culture Item 3 (MP)	.64	.24	.23
CQS (TP)	.13	−.14	.84
Openness to Experience (TP)	.13	.34	.67
Test of Personal Intelligence (TOPI) (MP)	.27	.64	-.02
Extraction method: Principal Component Analysis. Rotation method: Varimax with Kaiser Normalization. ^a^ Rotation converged in four iterations.			

Note: Three principal components had Eigenvalues greater than 1. Component 1 had an Eigenvalue of 3.91, accounting for 39.08% of the variance in the data. Component 2 had an Eigenvalue of 1.34, accounting for 13.43% of the variance in the data. Component 3 had an Eigenvalue of 1.12, accounting for 11.20% of the variance in the data. Cumulative percent variance accounted for was 63.71%.

**Table 7:** Rotated Principal Components Matrix ^a^: Psychometric assessments, SCIT Total with Psychometric Tests, VC, CQS, OE, and TOPI.

	Rotated Component		
	1	2	3
Letter Sets (MP)	.22	.86	.06
Figure Classification (MP)	.02	.78	.17
SCIT total (MP)	.73	.20	.06
Views-on-Culture Item 1 (MP)	.72	.27	.16
Views-on-Culture Item 2 (MP)	.82	−.02	.13
Views-on-Culture Item 3 (MP)	.75	.18	.13
CQS (TP)	.11	−.10	.89
Openness to Experience (TP)	.20	.32	.59
Test of Personal Intelligence (MP)	.38	.58	−.15
Extraction method: Principal Component Analysis. Rotation method: Varimax with Kaiser Normalization. ^a^ Rotation converged in five iterations.			

Note: Three principal components had Eigenvalues greater than 1. Component 1 had an Eigenvalue of 3.41, accounting for 37.94% of the variance in the data. Component 2 had an Eigenvalue of 1.30, accounting for 14.42% of the variance in the data. Component 3 had an Eigenvalue of 1.05, accounting for 11.62% of the variance in the data. Cumulative percent variance accounted for was 63.98%.

**Table 8:** Rotated Principal Components Matrix ^a^: Sternberg Cultural Intelligence Tests SCIT Business (SCIT-B) and Leisure (SCIT-L), Views-on-Culture; Cultural Intelligence Scale (CQS), Openness to Experience (OE), Test of Personal Intelligence (TOPI), SAT/ACT, and College GPA.

	Rotated Component			
	1	2	3	4
SCIT-B (MP)	.85	−.04	.06	−.02
SCIT-L (MP)	.91	.06	.04	−.06
Views-on-Culture Item 1 (MP)	.64	.29	.25	.10
Views-on-Culture Item 2 (MP)	.73	−.06	.14	.19
Views-on-Culture Item 3 (MP)	.68	.15	.08	.23
CQS (TP)	.15	.09	−.03	.88
Openness to Experience (TP)	.12	−.10	.67	.46
Test of Personal Intelligence (TOPI) (MP)	.18	.10	.85	−.20
SAT/ACT (MP)	−.09	.84	.09	.04
Cumulative college GPA (MP)	.23	.75	−.06	.02
Extraction method: Principal Component Analysis. Rotation method: Varimax with Kaiser Normalization. ^a^ Rotation converged in five iterations.				

Note: Four principal components had Eigenvalues greater than 1. Component 1 had an Eigenvalue of 3.47, accounting for 34.67% of the variance in the data. Component 2 had an Eigenvalue of 1.33, accounting for 13.42% of the variance in the data. Component 3 had an Eigenvalue of 1.13, accounting for 11.29% of the variance in the data. Component 4 had an Eigenvalue of 1.00, accounting for 10.04% of the variance in the data. Cumulative percent variance accounted for was 69.42%.

**Table 9:** Rotated Principal Components Matrix ^a^: SCIT, VC, CQS, OE, TOPI, SAT/ACT, and GPA.

	Rotated Component		
	1	2	3
SCIT total (MP)	.79	.00	.09
Views-on-Culture Item 1 (MP)	.69	.25	.23
Views-on-Culture Item 2 (MP)	.80	−.10	.13
Views-on-Culture Item 3 (MP)	.77	.11	.08
CQS (TP)	.30	.13	.20
Openness to Experience (TP)	.19	−.08	.75
Test of Personal Intelligence (TOPI)(MP)	.11	.08	.79
SAT/ACT (MP)	−.10	.87	.12
Cumulative college GPA (MP)	.28	.72	−.10
Extraction method: Principal Component Analysis. Rotation method: Varimax with Kaiser Normalization. ^a^ Rotation converged in four iterations.			

Note: Three principal components had Eigenvalues greater than 1. Component 1 had an Eigenvalue of 2.91, accounting for 32.36% of the variance in the data. Component 2 had an Eigenvalue of 1.32, accounting for 14.64% of the variance in the data. Component 3 had an Eigenvalue of 1.06, accounting for 11.75% of the variance in the data. Cumulative percent variance accounted for was 58.75%.

**Table 10:** Rotated Principal Factor Matrix: SCIT, VC, CQS, OE, TOPI, and SAT/ACT ^a^.

	Rotated Component		
	1	2	3
SCIT Total	.67	.03	.17
Views-on-Culture Item 1	.61	.21	.26
Views-on-Culture Item 2	.73	-.07	.14
Views-on-Culture Item 3	.68	.12	.12
CQS	.27	.10	.04
Openness to Experience	.26	-.03	.32
Test of Personal Intelligence	.11	.06	.74
SAT/ACT	-.06	.82	.07
Cumulative College GPA	.26	.43	-.03
Extraction method: Principal Component Analysis. Rotation method: Varimax with Kaiser Normalization. ^a^ Rotation converged in four iterations.			

Note: Three principal components had Eigenvalues greater than 1. Component 1 had an Eigenvalue of 2.91, accounting for 32.36% of the variance in the data. Component 2 had an Eigenvalue of 1.32, accounting for 14.64% of the variance in the data. Component 3 had an Eigenvalue of 1.06, accounting for 11.75% of the variance in the data. Cumulative percent variance accounted for was 58.75%.

## Data Availability

Data are available from coauthor Chak Haang Wong, cw574@cornell.edu. The SCIT is available from Robert J. Sternberg, robert.sternberg@cornell.edu.
